# A 20‐year scoping review of the veterinary interventional radiology and interventional endoscopy literature (2000‐2019)

**DOI:** 10.1111/jvim.16748

**Published:** 2023-05-26

**Authors:** Nina Samuel, Michelle A. Giuffrida, William T. N. Culp, Carrie A. Palm

**Affiliations:** ^1^ William R. Pritchard Veterinary Medical Teaching Hospital University of California‐Davis Davis California USA; ^2^ Department of Surgical and Radiological Sciences, School of Veterinary Medicine University of California‐Davis Davis California USA; ^3^ Department of Medicine and Epidemiology, School of Veterinary Medicine University of California‐Davis Davis California USA

**Keywords:** evidence‐based medicine, veterinary, minimally invasive, therapy, veterinary

## Abstract

**Background:**

Interventional radiology (IR) and interventional endoscopy (IE) have broad potential for minimally invasive therapy in veterinary patients, but the scope of original peer‐reviewed veterinary IR/IE research publications has not been described.

**Objectives:**

Catalogue published applications and indications for noncardiac therapeutic IR/IE in animals and describe type and quality of veterinary IR/IE research over 20 years.

**Methods:**

Highly‐cited veterinary journals were searched to identify articles published 2000 to 2019 involving therapeutic IR/IE applications for clinical veterinary patients. Articles were assigned a level of evidence (LOE) according to published standards. Authorship, animal data, study design, and interventions were described. Change in publication rate, study size, and LOE of IR/IE articles over time was analyzed.

**Results:**

One hundred fifty‐nine of 15 512 (1%) articles were eligible, including 2972 animals. All studies were low LOE and 43% were case reports with ≤5 animals. Number of IR/IE articles per year (*P* < .001), proportion of journals' articles pertaining to IR/IE (*P* = .02), and study size (*P* = .04) all increased over time, but LOE (*P* = .07) did not. Common target body systems were urinary (40%), digestive (23%) respiratory (20%), and vascular (13%). Common indications were nonvascular luminal obstructions (47%), object retrieval (14%), and congenital anomalies (13%). Most procedures involved indwelling medical devices or embolic agents, whereas tissue resection and other procedures were less common. Procedures utilized fluoroscopy (43%), endoscopy (33%), ultrasound (8%), digital radiography (1%), or fluoroscopy in combination with other modalities (16%).

**Conclusions:**

Treatments involving IR/IE have wide applicability in veterinary medicine but large, rigorous, and comparative studies describing these procedures are lacking.

**Abbreviations**
BMCVR: BMC Veterinary Research
CI: confidence interval
EVJ: Equine Veterinary Journal
FVS: Frontiers in Veterinary Science
IE: interventional endoscopy
IR: interventional radiology
JAVMA: Journal of the American Veterinary Medical Association
JFMS: Journal of Feline Medicine and Surgery
JSAP: Journal of Small Animal Practice
JVECC: Journal of Veterinary Emergency and Critical Care
JVIM: Journal of Veterinary Internal Medicine
LOE: level of evidence
RVS: Research in Veterinary Science
VJ: The Veterinary Journal
VS: Veterinary Surgery

## 1 INTRODUCTION

Interventional radiology (IR) and interventional endoscopy (IE) use contemporary imaging modalities such as fluoroscopy and endoscopy to allow for minimally invasive treatment of benign and malignant diseases in people and animals.^1^, ^2^, ^3^, ^4^, ^5^, ^6^, ^7^, ^8^ Potential veterinary therapeutic applications are broad and include targeted delivery of medications, placement of medical devices, enlargement of luminal stenoses or constrictions, and retrieval of foreign objects and calculi, among others.^1^, ^2^, ^3^, ^4^, ^7^, ^8^ Proposed advantages of IR/IE include preservation of anatomy and physiologic function and decreased morbidity relative to what might be expected with open surgical procedures, as well as ability to facilitate treatment in disease situations that lack other traditional options.^3^, ^4^, ^7^ Minimally invasive IR/IE methods are used extensively in human medicine and are considered the gold standard for treating disease conditions such as small‐ to moderately sized tumors where tissue sparing is desired (eg, renal, liver, and lung tumors and metastases),^9^ certain nonresectable tumors,^10^ ureteral obstruction,^11^ vascular anomalies,^12^ and portal hypertension.^13^

In veterinary medicine, IR treatment for congenital and acquired cardiac disease has been widely reported and practiced during the past two decades,^14^ whereas the applications and evidence base of the noncardiac IR/IE literature has not been cataloged. In our experience, noncardiac IR/IE techniques span different disciplines and similar applications might be performed by a surgeon, internist, or criticalist rather than existing under a singular subspecialty. This cross‐disciplinary reach could obscure the extent to which techniques have gained extensive application or undergone rigorous clinical or experimental evaluation. Because of the broad potential applications and benefits of IR/IE techniques in veterinary patients, attention should be directed to the current evidence available and to gaps in knowledge to broaden clinical use and research in the field.

Our purpose was to conduct a scoping review of original scientific articles pertaining to noncardiac veterinary IR and IE published in 11 prominent peer‐reviewed journals over a 20‐year period. Our objectives were to describe applications and indications for published IR/IE procedures, gain insight into the extent to which veterinary IR/IE is practiced or researched, and gauge the general level of evidence (LOE) of veterinary research in this area.

## 2 MATERIALS AND METHODS

Original research reports published in 11 prominent peer‐reviewed English language scientific journals—*BMC Veterinary Research* (BMCVR), *Equine Veterinary Journal* (EVJ), *Frontiers in Veterinary Science* (FVS), *Journal of the American Veterinary Medical Association* (JAVMA), *Journal of Feline Medicine and Surgery* (JFMS), *Journal of Veterinary Emergency and Critical Care* (JVECC), *Journal of Veterinary Internal Medicine* (JVIM), *Journal of Small Animal Practice* (JSAP), *Research in Veterinary Science* (RVS), *The Veterinary Journal* (VJ), and *Veterinary Surgery* (VS)—from 2000 to 2019 were screened for inclusion. The journals were selected for review from the Journal Citation Reports Veterinary Sciences Edition (Clarviate Analytics, 2022) database of 169 publications. Eligible journals were subject to the following criteria: (a) minimum 4000 total citations to the journal's articles during publication history, (b) journal impact factor in the top 2 quartiles (top 50%) of journals in the Veterinary Science category, (c) journal scope includes original research pertaining to clinical application of treatment in privately owned individual animals, and (d) English language. Journals meeting these criteria were further screened by review of every article published in the years 2000, 2001, 2002, 2017, 2018, and 2019. Two journals (*Frontiers in Veterinary Sciences* and *BMC Veterinary Research*) originated after 2002, and the first 3 years of each journal's publication history was screened in lieu of 2000 to 2002. If no eligible IR/IE article was identified in the 6‐year screening period, the journal was excluded from the study. If at least one eligible IR/IE article was identified during the 6‐year screening period, the journal was included in the study. Eligible articles were original research reports in which a study objective involved describing, comparing, or otherwise evaluating a therapeutic IR or IE technique intended for use in clinical veterinary patients. Table 1 describes the definitions of IR/IE and types of included and excluded interventions used for the purposes of this review. Interventional orthopedic and computer‐assisted surgical applications were excluded. Articles relating to treatments applied to the heart muscle, valves, or immediate inflow or outflow vasculature were excluded. Editorials, book reviews, narrative review articles, and studies in which IR/IE occurred but was not central to study objectives were excluded. An example of an article excluded on the latter basis would be a study of urinary cancer chemotherapy outcomes in which some animals were treated with stents but the stent technique or outcomes were not specifically addressed. Articles in which IR/IE techniques were applied with the aid of open surgery were retained.

**TABLE 1:** Definitions used for eligibility criteria.

Category	Definition	Includes	Does not include
Therapeutic interventional radiology	Minimally invasive therapeutic procedures performed using medical imaging guidance, such as fluoroscopy, computed tomography, magnetic resonance imaging, or ultrasound	Medical device placement Object retrieval	Purely diagnostic procedures such as cystourethrogram
		Targeted injection of medical substances	Use of imaging purely as an aid to open surgery, such as to identify anatomy or check position of implants placed in open fashion
		Tissue ablation	Thoracoscopy, laparoscopy, arthroscopy, and other camera‐based procedures
			Robotic or computer‐aided navigation or surgery
Therapeutic interventional endoscopy	Minimally invasive therapeutic procedures performed using guidance of cameras inserted into natural body orifices or into mucosal passages with the aid of a surgical approach	Dilation	Purely diagnostic procedures such as biopsy, visualization of anatomy
		Hemostasis	
		Implantation of medical tubes and devices	
		Object retrieval	Thoracoscopy, laparoscopy, arthroscopy, and other camera‐based procedures involving body cavities and nonmucosal spaces
		Targeted injection of medical substances	
		Tissue ablation or excision	

A single investigator performed an online manual search of titles, abstracts, and full texts to screen each journal issue for potentially eligible articles. For each article identified, two investigators independently read title, abstract and full text to determine whether the article met study inclusion criteria, discussed their results, and resolved discrepancies by joint review and discussion. Data charting was performed by a single investigator using a form jointly developed by two investigators including publication year and journal, first and corresponding author names and institutional affiliations, country where the research was performed, study design, species, number of animals studied, body system to which the intervention was applied, type of intervention, and indication for intervention. Each article was assessed for the presence of specific methodologic features and assigned a LOE according to guidelines published by the Oxford Centre for Evidence‐Based Medicine.^15^ Single case reports, case series with ≤5 animals, and preclinical studies such as those involving research animals or cadaver tissues were rated level 5 evidence. Levels of evidence rated 1 or 2 were considered “high” level evidence and LOE rated 3, 4, or 5 were considered “low” level evidence.^16^

Summary statistics were used to convey descriptive data. Simple linear regression was used to test the null hypotheses of no linear relationship between advancing year and the following predictor variables: annual number of IR/IE articles published, annual proportion of total journal articles pertaining to IR/IE, and number of study subjects (n) per IR/IE article. The nonparametric Cuzick test for trend using ranks was used to evaluate the association between LOE of IR/IE articles (graded 1‐5, where 1 is highest LOE and 5 is lowest LOE) and publication year. Tests were 2‐sided and *P* < .05 was considered statistically significant.

## 3 RESULTS

**FIGURE 1:**
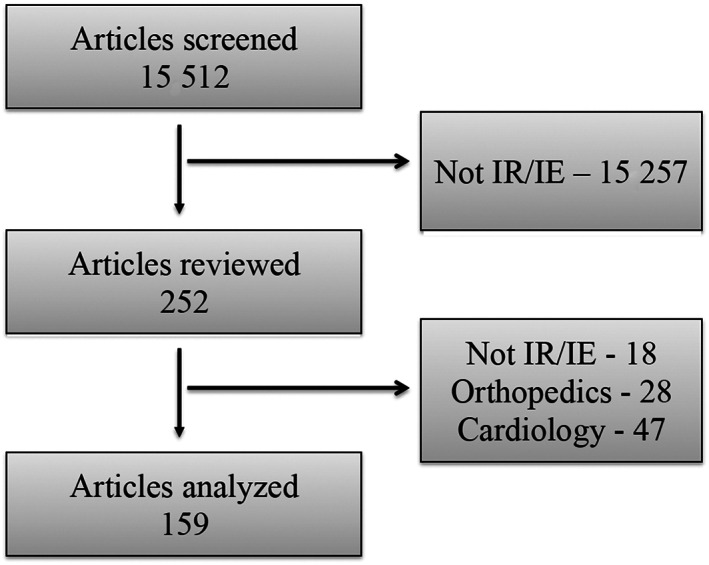
Search flow identifying 159 eligible veterinary IR/IE articles.

Among 11 journals screened using the 6‐year review protocol, 4 did not publish any IR/IE articles during the 6 years and were excluded from the study (Table 2). Among the remaining 7 journals, the search identified 159 eligible articles among 15 512 original research articles (Figure 1), representing 1.0% of the articles published during the 20‐year period. The mean number of IR/IE articles published per year was 9.2 (SD, 3.7; range, 3‐17) and accounted for a mean of 1.4% (SD, 0.6; range, 0.6‐2.7) of all original articles published in the 7 journals. Both total number of IR/IE articles and proportion of journals' articles pertaining to IR/IE increased over time. Positive linear associations were found between publication year and number (*β* = 0.58; 95% confidence interval [CI], 0.33 to 0.82; *P* < .001) and proportion (*β* = 0.05; 95% CI, −0.01 to 0.10; *P* = .02) of IR/IE articles over the 20‐year study period.

**TABLE 2:** Journals and articles screened and selected for study inclusion.

Journal^a^	Screened journals		Included journals		
	Articles screened (6 years)	IR/IE articles (6 years)	Articles screened (20 years)	IR/IE articles (20 years)	% IR/IE articles (20 years)
BMCVR	1376	0	—	—	
EVJ	606	2	2060	3	0.1%
FVS	841	0	—	—	
JAVMA	1092	34	3591	83	2.3%
JFMS	419	1	1517	11	0.7%
JVECC	250	3	887	7	
JVIM	931	7	3352	22	0.7%
JSAP	520	6	1836	14	0.8%
RVS	869	0	—	—	
VJ	515	0	—	—	
VS	605	7	2269	19	0.8%
Total			15 512	159	1.0%

^a^ BMCVR, *BMC Veterinary Research*; EVJ, *Equine Veterinary Journal*; FVS, *Frontiers in Veterinary Science*; JAVMA, *Journal of the American Veterinary Medical Association*; JFMS, *Journal of Feline Medicine and Surgery*; JVECC, *Journal of Veterinary Emergency and Critical Care*; JVIM, *Journal of Veterinary Internal Medicine*; JSAP, *Journal of Small Animal Practice*; RVS, *Research in Veterinary Science*; VJ, *The Veterinary Journal*; VS, *Veterinary Surgery*.

Research originated primarily at academic institutions in the United States (86/159, 54.1%) or abroad (30/159, 18.9%). The remaining 43 articles reflected research performed at private hospitals (n = 31) or a combination of private and academic settings (n = 12). Although 59 different institutions were represented, 65/159 (40.9%) of the articles were affiliated with three institutions: the Animal Medical Center in New York, NY (n = 25), the University of California, Davis (n = 20), and the University of Pennsylvania (n = 20). No other institution was associated with more than five articles.

Species studied included dogs (89/159, 56.0%), cats (35/159, 22.0%), horses (20/159, 12.6%), multiple species (12/159, 7.6%), ferret (1/159, 0.6%), cow (1/159, 0.6%), and tortoise (1/159, 0.6%). The majority of articles (147/159, 92.5%) were clinical in nature and involved IR/IE applied to privately owned animals with naturally occurring disease conditions. The remaining 12 articles described pre‐clinical proof‐of‐concept studies involving cadaver tissues or research animals. No articles were classified as high level evidence. One of 159 (0.6%) articles was classified as level 3 evidence, 77/159 (48.4%) articles were classified as level 4 evidence, and 81/159 (50.9%) were classified as level 5 (lowest) evidence. Among 147 clinical articles, the following study designs were represented: retrospective descriptive study of >5 animals (59/159, 40.1%), single case report (47/159, 32.0%), case report of 2 to 5 animals (22/159, 15.0%), prospective descriptive study (12/159, 8.2%), retrospective comparative study (6/159, 4.1%), prospective nonrandomized comparative study (1/159, 0.6%), and randomized controlled trial (1/159, 0.6%). Overall, 19/159 (12.0%) articles were prospective and 140/159 (89.0%) were retrospective. Clinical articles included data for 2972 animals; the median number of animals per study was 7 (IQR, 1‐25; range, 1‐193). The number of subjects per article increased over time, but LOE did not. A positive linear association was found between number of subjects per article and publication year (*β* = 0.82; 95% CI, 0.04 to 1.61; *P* = .04) and no association was found between LOE grade of published articles and publication year (*P* = .07).

Research involved therapeutic procedures classified as IR in 87/159 (54.7%) articles, as IE in 53/159 (33.3%) and as both IR and IE in 19/159 (12.0%) articles. In the 87 articles classified as IR, imaging modalities utilized to perform IR procedures were fluoroscopy alone (68/87, 78.2%), ultrasound alone (13/87, 14.9%), digital radiography alone (1/87, 1.1%), or a combination of fluoroscopy and ultrasound (5/87, 5.7%). Among 53 articles classified as IE, 52 (98.1%) used utilized flexible or rigid endoscopy, or both, and 1 article (1.9%) described endoscopy and fluoroscopy. In the 19 articles classified as both IR and IE, both fluoroscopy and endoscopy were used to perform all procedures.

Target body systems for IR/IE procedures were urinary (64/159, 40.3%), digestive (36/159, 22.6%), respiratory (31/159, 19.5%), vascular (21/159, 13.2%), endocrine (3/159, 1.9%), ocular (2/159, 1.3%), musculoskeletal (1/159, 0.6%), and multiple (1/159, 0.6%). General indications for IR/IE procedures were nonvascular luminal obstruction (75/159, 47.2%), object retrieval (22/159, 13.8%), congenital anomaly (21/159, 13.2%), persistent hemorrhage (13/159, 8.2%), solid neoplasia (12/159, 7.6%), feeding tube placement (4/149, 2.5%), prevention of gastric volvulus (3/159, 1.9%), endocrinopathy (3/159, 1.9%), pyonephrosis (2/159, 1.3%), and other conditions including laryngeal hemiplegia (1/159, 0.6%), soft tissue injury (1/159, 0.6%), navicular disease (1/159, 0.6%), and pleural space disease (1/159, 0.6%).

The majority (92/159, 57.9%) of interventional procedures involved placement of indwelling medical devices (eg, stents, diversion tubes or systems, embolic coils) either as a sole procedure (n = 87) or in combination with other procedures (n = 5). Other types of procedures included resection or ablation of tissues (25/159, 15.7%), removal of calculi with or without the aid of lithotripsy (11/159, 6.9%), infusion of medical substances (eg, ethanol, tissue plasminogen activator, collagen, carboplatin) into tissues or blood vessels (11/159, 6.9%), retrieval of foreign objects or medical devices (10/159, 6.3%), dilatation of lumens using balloons or bougies (7/159, 4.4%), device placement in combination with ≥1 of the above (5/159, 3.1%), thrombectomy (1/159, 0.6%), and abscess drainage (1/159, 0.6%). One article described the use of a specific IR technique to gain anatomic access for the subsequent application of various other IR/IE procedures including device placement, tissue ablation, and infusion.

Articles involving medical device placement described the application of intraluminal stents (43/92, 46.7%), embolic agents or devices (26/92, 26.3%), diversion catheters or tubes (17/92, 18.5%), T‐fasteners (3/92, 3.3%), or both stents and diversion catheters (3/92, 3.3%).

Among 64 articles describing therapeutic procedures involving the urinary tract, 29 (45.3%) were classified as IR, 24 (37.5%) were classified as IE, and 11 (17.2%) used a combination of IR and IE techniques. Surgery was utilized for access or to complete therapeutic procedures in 19 (29.7%) studies. The most common clinical scenario involved placement of stents or urinary diversion systems (eg, subcutaneous ureteral bypass, cystostomy tube) for the relief of ureteral or urethral obstructions attributed to neoplasia or benign causes such as calculi and strictures (31/64, 48.4%). Other common clinical scenarios involved removal of urinary calculi with or without the aid of laser or electrohydraulic lithotripsy (10/64, 15.6%) and application of electrosurgery or laser to resect obstructive neoplasia or correct congenital anomalies (eg, ectopic ureter, ureterocele, ureterovesicular stenosis) of the lower urinary tract (10/64, 15.6%). Other clinical applications were sclerotherapy for idiopathic renal hematuria (2/64, 3.1%), endoscopic‐guided injection of urethral bulking agents (2/64, 3.1%), renal pelvic lavage and ureteral stent placement for treatment of obstructive pyonephrosis (1/64, 1.6%), targeted intravascular chemotherapy of lower urinary tract neoplasia (1/64, 1.6%), laser ablation of hemorrhagic lesions (1/64, 1.6%), balloon dilatation of lower urinary tract neoplasia (1/64, 1.6%), percutaneous renal abscess drainage (1/64, 1.6%), renal pelvic ethylenediaminetetraacetic acid infusion to treat implant encrustation (1/64, 1.6%), application of embolic agents to treat urethral duplication (1/64, 1.6%), and laser lithotripsy to remove an encrusted ureteral stent (1/64, 1.6%). One article (1.6%) described a percutaneous perineal approach to perform various endourologic procedures in male dogs.

Among 36 articles describing therapeutic procedures involving the digestive system, 20 (55.6%) were classified as IE, 13 (36.1%) were classified as IR, and 3 (8.3%) used both IR and IE techniques. The most common clinical scenarios were application of electrosurgery or laser alone or in combination with a snare to resect intraluminal tissues (6/36, 16.7%), retrieval of foreign objects or medical devices (6/36, 16.7%), intraluminal stent placement to alleviate benign or malignant obstructions of the colon or esophagus (5/36, 13.9%), balloon dilatation or bougienage for benign esophageal or pharyngeal strictures (4/36, 11.1%), placement of indwelling feeding tubes (4/36 11.1%), application of percutaneous gastropexy devices to prevent gastric dilatation volvulus (3/36, 8.3%) and arterial embolization for treatment of hepatic tumors (3/36, 8.3%). Other clinical applications included alleviation of extrahepatic biliary obstruction via percutaneous cholecystostomy tube (1/36, 2.8%) or endoscopic biliary stent (1/36, 2.8%), placement of an indwelling balloon esophagostomy tube (1/36 2.8%), retrieval of an esophageal foreign body with subsequent intraluminal stent placement alone (1/36, 2.8%) or in combination with balloon dilatation (1/36, 2.8%).

Among 32 articles describing therapeutic procedures involving the respiratory system, 19 (59.4%) were classified as IR, 9 (28.1%) were classified as IE, and 4 (12.5%) used a combination of IR and IE techniques. The most common clinical scenarios were placement of intraluminal stents to alleviate airway obstruction (11/32, 34.4%) and placement of embolic agents to alleviate upper respiratory hemorrhage (9/32, 28.1%). Other clinical applications included transendoscopic use of electrocautery, electrosurgery, or laser to resect tissue in the larynx, trachea, or lower airways (6/32, 28.1%) and retrieval of intraluminal foreign material (2/32, 6.3%), percutaneous chest tube placement (1/32, 3.2%) and balloon dilatation of benign nasopharyngeal stenosis either alone (1/32, 3.2%) or in combination with stent placement (1/32, 3.2%).

Among 20 articles describing therapeutic procedures involving the vascular system, all were classified as IR. The most common clinical scenario was embolization of portosystemic shunts (6/20, 30.0%) or arteriovenous malformations or fistulae (4/20, 20.0%). Two articles describe intravascular stents for treatment of Budd‐Chiari‐like syndrome. Other applications described in one article each (5.0%) were embolization of a pseudoaneurysm, embolization of a varix, placement of a vascular stent to alleviate mass‐associated chylothorax, retrieval of an intravascular foreign body, placement of IV catheters, placement of a vascular closure device in a dog with a bleeding disorder, intra‐arterial stem cell injection, and balloon thrombectomy of an aorto‐iliac‐femoral thrombosis.

Of the remaining articles, two described percutaneous ethanol ablation of thyroid tissue, one described percutaneous heat ablation of parathyroid tissue, one1 described nasolacrimal stent placement, one described navicular bursa injections, one described removal of an ocular foreign body, and one described embolization or chemoembolization of solid tumors in multiple body systems.

## 4 DISCUSSION

We documented that over the past two decades, thousands of domestic animals were treated using therapeutic IR and IE techniques for dozens of different indications across all major body systems. Publication in the field increased modestly over time with a substantial minority of publications attributed to a few institutions, suggesting that research involving therapeutic IR/IE remains a niche area despite its diverse applicability. The body of literature consisted primarily of retrospective case reports and case series, with few comparative studies and none with high LOE, although the number of animals per article demonstrated linear improvements over time.

Ample evidence exists in human and veterinary medicine demonstrating the benefits of minimally invasive treatments and procedures. These include less pain, lower morbidity, decreased anesthesia time, shorter hospital stays, and more rapid return to function compared to open surgical procedures that achieve similar therapeutic results.^7^, ^17^, ^18^, ^19^ In our experience, most veterinary IR/IE techniques are used in situations in which effective alternative treatments exist but are associated with less than optimal risk profiles or invasive surgical approaches. Although our data does not directly address motivations for applying IR/IE in veterinary patients, the indications for use such as foreign body retrieval and correction of congenital anomalies are consistent with intent to decrease morbidity, improve treatment precision, and shorten the duration of recovery relative to what is expected with open surgery. Other indications such as obstructive urinary tumors, idiopathic renal hemorrhage, extensive hepatic tumors, and intrathoracic tracheal collapse do not have effective medical or surgical options that also would preserve organ function, leading us to conclude that IR/IE was performed because surgery was not considered feasible or would carry unacceptable risk or loss of function. Development, research, and publication of novel procedures that can improve options, outcomes, and quality of life are critical to our profession's obligation to provide the best care to animal patients.

Although we observed an increasing number of IR/IE publications over the 20‐year time span, the overall evidence base remains limited. In the journals reviewed, the quality of the published IR/IE evidence was low according to the hierarchical scheme we applied, with no high‐quality comparative studies. Retrospective case reports and case series make up a large proportion of the veterinary clinical literature^20^, ^21^ and thus it is not surprising to find this pattern manifested in the IR/IE literature. There are many reasons why original IR/IE articles might have a particular predilection toward descriptive case series. Interventional treatments involve costly equipment, specialized training and practice, and careful case selection that can limit opportunities for case accrual and large prospective studies. Animal owners might not choose IR/IE procedures that are more expensive than effective standard of care treatments, or that primarily offer palliation for end‐of‐life situations. Many IR/IE techniques are applied in situations where case standardization is challenging or impossible and it can be difficult to attribute outcomes to treatment vs underlying disease. In other settings, the role of IR/IE is to create an opportunity for treatment where none previously existed, such that animals cannot ethically be randomized to receive no care. The practice of many IR/IE techniques can be very patient‐specific, delivering individualized care based on that animal's specific anatomy. Although this feature is a major strength of IR/IE treatment, it also means that the research is highly clinical in nature and focused on clinical reports and outcomes. Although this review evaluated 20 years of literature, it takes time for new treatments to be developed, reported, and incorporated into clinical practice and study.

A small number of institutions contributed a substantial minority of the articles identified in our review, with no other institution generating more than a few IR/IE studies over the 20 years. Possible explanations for this finding include a relative lack of resource allocation toward dedicated IR/IE programs by veterinary research institutions and funding agencies, a focus on clinical practice rather than research by IR/IE practitioners, or a small number of IR/IE practitioners. To our knowledge, IR and IE are not formally recognized subspecialty areas by any veterinary specialty college or equivalent veterinary professional organization, and we are unaware of formal clinical or research training programs with an exclusive IR/IE focus. These factors could limit the extent to which new clinical expertise and research centers in the field are generated.

The limitations of our study primarily pertain to potential selection bias at the levels of search and review. We limited our review to 11 English language veterinary journals but it is likely we could have identified more articles using a wider manual search strategy of more titles. Alternatively, we might have identified a larger number of sources by entering a limited number of relevant terms in a large database, but the lack of standardized terminology or medical subjects headings and wide variety of procedures and diseases led us to avoid a database strategy. Two investigators reviewed each journal issue manually to identify eligible articles and it is possible that some relevant articles were missed if IR/IE procedures were poorly described, constituted a minor part of the article, or were simply overlooked by both investigators. We did not contact journals to determine if any policies existed during 2000 to 2019 with regard to the publication of IR/IE content, case reports, or study sample sizes. Therefore, it is possible that observed publication patterns could reflect editorial policy or publication bias rather than the generation of content at the investigator level.

Our scoping review highlights for the first time the wide application of IR/IE in veterinary medicine and the thousands of animals that have received these treatments. The body of evidence however is lacking large, rigorous, and comparative studies, and many publications are attributed to a small number of institutions.

## CONFLICT OF INTEREST DECLARATION

Authors declare no conflict of interest.

## OFF‐LABEL ANTIMICROBIAL DECLARATION

Authors declare no off‐label use of antimicrobials.

## INSTITUTIONAL ANIMAL CARE AND USE COMMITTEE (IACUC) OR OTHER APPROVAL DECLARATION

Authors declare no IACUC or other approval was needed.

## HUMAN ETHICS APPROVAL DECLARATION

Authors declare human ethics approval was not needed for this study.
